# Artificial Intelligence-Generated Electronic Medical Record Summarization in Breast Surgical Oncology

**DOI:** 10.1245/s10434-026-20170-w

**Published:** 2026-07-15

**Authors:** Ko Un Park, Bergen K. Sather, Anika Shah, Christina A. Minami, Matthew Butler, Lara Butler, Jordan A. Love, Kathleen McLean, Adam Dunn, Elizabeth A. Mittendorf, Tari A. King

**Affiliations:** 1 Division of Breast Surgery, Department of Surgery, Brigham and Women’s Hospital, Boston, MA USA; 2 Ariadne Labs, Brigham and Women’s Hospital, Harvard T.H. Chan School of Public Health, Boston, MA USA; 3 Present Address: Breast Oncology Program, Dana-Farber Cancer Institute, Boston, MA USA; 4 Harvard Medical School, Boston, MA USA; 5 Department of General and Thoracic Surgery, Virginia Mason Medical Center, Seattle, WA USA; 6 Present Address: Department of Surgery, Foothills Medical Centre, Cumming School of Medicine, University of Calgary, Alberta, Canada; 7 Present Address: Brown University Health, Providence, RI USA; 8 Present Address: Division of Breast Surgery, Department of Surgery, Beth Israel Deaconess Medical Center, Boston, MA USA; 9 Present Address: Winship Cancer Institute of Emory University, Atlanta, GA USA

**Keywords:** Artificial intelligence, Large language model, Medical record summarization, Breast surgical oncology, PDSQI-9

## Abstract

**Background:**

Reviewing pathology, imaging, and consultation documents in oncology can be time-consuming, particularly when records originate from external facilities in different file formats. This study aimed to evaluate the impact of a Retrieval-Augmented Generation (RAG)-enabled GPT-4o summarization agent on clinical workflows and quality of outside-record summaries in breast surgical oncology.

**Methods:**

Initial performance evaluation of a GPT-4o/RAG agent to generate summaries of oncologic reports in 50 charts followed by a prospective pilot test of sequential cases, with each AI summary evaluated using a modified Provider Documentation Summarization Quality Instrument (PDSQI-9; 1–5 Likert scale), including dichotomized ratings (low [1–3], high [4, 5]), binomial testing, frequency and type of user-reported errors, clinician-coded error criticality (treatment-impacting vs noncritical). Pre- and post-use survey of documentation burden (NASA TLX) and user experience was performed.

**Results:**

Among 62 cases, AI-generated summaries were rated high for accuracy, usefulness, succinctness, and source citation. Thoroughness without omission was rated low in 28 (45%) summaries. Errors were noted in 25 (40%) surveys, with 13 (52%) classified as critical (treatment-impacting). The most common error type involved imaging, reported in 17 (68%) cases. For perceived time savings, the median response was neutral, but qualitative feedback described the tool as helpful for straightforward cases and as reducing typing burden but requiring workflow adjustment and improvements for complex cases.

**Conclusions:**

Although users rated RAG-enabled GPT-4o agent-generated documentation summaries favorably on several quality domains, they frequently lacked thoroughness and occasionally contained treatment-relevant errors. Human review and further iteration of the technology remain necessary before implementation.

**Supplementary Information:**

The online version contains supplementary material available at https://doi.org/10.1245/s10434-026-20170-w.

Effective oncology care coordination hinges on timely access to and accurate summarization of oncology information. Review of clinical documentation before consultation (pre-charting), including medical, radiology, pathology, and genetic reports, can be time-consuming, especially when care was received at an external institution with a different electronic health record (EHR) system. Pre-charting is a critical task in ambulatory oncology care that requires navigation of various sections of the EHR to review and summarize pertinent parts of the patient’s medical record. Variability in documentation formats (e.g., fax reports, PDF, text files, e-mails) adds significant administrative and cognitive burden for clinical teams and presents an opportunity to leverage generative artificial intelligence (AI) systems such as large language models (LLMs).

The surgical management of breast cancer specifically depends on the precise integration of several information sources including multiple detailed imaging methods (mammogram, ultrasound, MRI) and often more than a single pathologic assessment. Small discrepancies or omissions in medical record review can alter treatment recommendations significantly, thus making breast surgical oncology a high-stakes domain for summarization needs.

Studies have investigated AI generated summarization as an approach to reduce information overload in clinical settings by extracting patient data and organizing it into concise, structured outputs.^1^ However, these summarization systems face implementation challenges including temporal abstraction, redundancy, factual consistency, and hallucinations, highlighting the need for careful validation and human oversight.^2,3^

To address the need for breast surgical oncology-specific, structured, and clinically reliable summaries, we developed a secure, AI agent for chart summarization. This study aimed to evaluate the accuracy, feasibility, and acceptability of the AI-generated summaries and to understand the user experience with AI-generated summaries.

## Methods

### AI Model Development

**Fig. 1:**
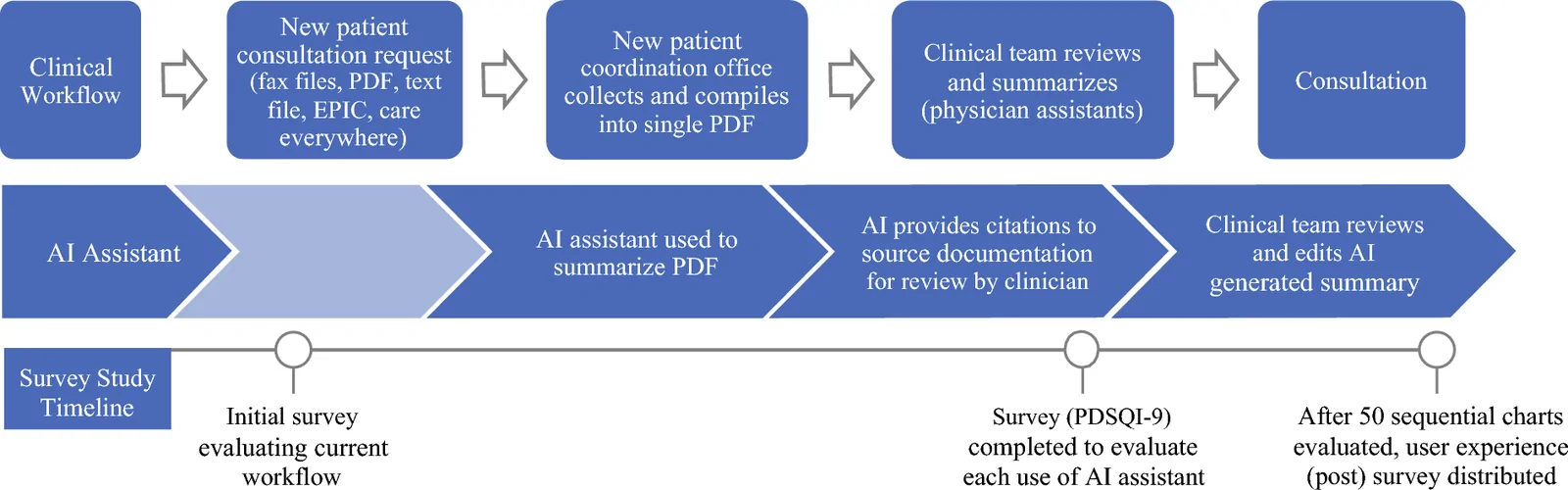
Pilot testing of a workflow diagram with current clinical workflow, AI integration, and survey study timeline

As part of usual workflow, new patient radiology, pathology, and relevant external consultation reports were compiled into PDF files by record specialists (Fig. 1). The PDF included reports maintained in their original formats, representing significant variability from various institutions. These PDF files then were processed using a retrieval-augmented generation (RAG) approach.

The RAG approach combines information retrieval and text generation by grounding the system on a specific knowledge source such that the system extracts pertinent information efficiently.^4^ To achieve this, we used vectorization and embedding techniques to convert textual data into numeric representations. This enabled the AI search function to effectively identify and extract key topics from the documents. The extracted information then was used to generate history of present illness (HPI) summaries using the generative AI GPT-4o model in an institution-secured platform. The development of the platform involved prompt engineering, with specific prompts designed to guide AI in generating relevant summaries. The model was developed to provide “citations” (file name and page number) from which the information was retrieved. Data were reported according to the TRIPOD-LLM guidelines.^5^

### Initial AI Model Evaluation

Four breast surgery physician assistants (PAs) generated 50 HPI summaries and compared them with the AI-generated HPIs of identical records. The accuracy of the output was tested item-by-item by two breast oncology specialists (K.P. and C.M.). The AI-generated HPIs were clinically validated by comparisons with the human HPIs, using a structured evaluation framework that focused on accuracy (factually correct/incorrect) and completeness of information (“complete” if the AI response aligned with the key information that oncologists would anticipate in a human-generated summary) across the following predefined domains: patient age, imaging findings (mammogram, ultrasound, MRI, biopsy modality), pathology findings (histology, receptor status), medical history, and pending workup. The organization of the information (clarity) and contextual relevance of the information (relevance) were rated on a 5-point Likert scale with a range of 1 (strongly disagree) to 5 (strongly agree). Interpretation of mean scores was based on the scale-range formula, with mean scores categorized as low (1.00–2.33), moderate (2.34–3.66), and high (3.67–5.00).^6^

### Pilot Testing

A pilot study was conducted in September 2025 to characterize existing clinical documentation practices and associated cognitive burden (pre-survey), evaluate the quality of AI-generated HPI summaries (modified Provider Documentation Summarization Quality Instrument [PDSQI-9]), and collect feedback on user experience with the AI assistant (post-survey) (Fig. 1). Members of the breast surgery clinical team (PAs and breast surgeons) were provided with instructions on how to use the AI assistant. Eligible patients were identified as those whose initial breast cancer workup was performed at an external institution with medical records scanned as a PDF (rather than direct input into the institution’s EHR system). As part of usual workflow, relevant oncology records were collected and compiled as a PDF by the new patient office. Typically, the PAs summarize the PDF information in an HPI before the patient’s consultation with the surgeon. We did not specify whether the AI summary should be generated before or after the human HPI.

The initial (pre-)survey assessed current clinical documentation practices. It included an abbreviated version of the NASA Task Load Index (NASA TLX), specifically focusing on the mental and temporal demand subscales as a surrogate for documentation burden.^7–9^ Each subscale was rated on a 20-point scale to capture perceived workload demand. Higher scores represent a higher workload demand.

To rate the quality of each AI-generated summary, a modified PDSQI-9 was administered to the providers (Supplemental File). The PDSQI-9, scored on a 5-point Likert scale, is a validated instrument for evaluating LLM-generated clinical summaries with high inter-rater reliability.^10^ To reduce respondent survey burden and avoid redundancy in previous evaluation of the model, three (organization, comprehensibility, and need for abstraction) of the nine original PDSQI-9 attributes were omitted from the survey. These concepts had overlap with the initial AI model evaluation, in which organization was evaluated directly, abstraction was evaluated within completeness and contextual relevance, and comprehensibility was assessed through organizational clarity and accuracy. Each PDSQI-9 attribute was analyzed independently as a single-item measure, consistent with prior studies demonstrating that independent survey items can provide meaningful information when assessing distinct constructs.^11^

The post-survey was administered to assess overall user perspectives after the AI assistant had been used in 62 charts. The survey included questions regarding frequency of AI assistant use if the AI assistant saved time (5-point Likert scale), free text comments about end-user experience, and mental and temporal demand subscales from the NASA TLX workload instrument. The study was approved by the Mass General Brigham Institutional Review Board.

### Statistical Analysis

Simple description and comparison of means were analyzed using data collected from the respondents. The NASA TLX scores were reported as means, and Welch’s *t* test was performed to compare pre- and post-AI assistant introduction scores. Consistent with established recommendations for analyzing Likert data, PDSQI-9 Likert ratings were reported as means and dichotomized a priori into collapsing categories with 1 to 3 as “low” and 4 to 5 as “high.” The midpoint was included in negative to reflect absence of strong endorsement and analyzed with binomial tests (*p* < 0.05). Likert scores for the post-survey were classified as disagreement (1–2), neutral (3), or agreement (4–5). Free-text responses were coded by error type and criticality (treatment-impacting vs non-critical) by two clinicians.

## Results

### Initial AI Model Evaluation

The AI model was evaluated for clinical accuracy and completeness in 50 charts before pilot testing. The AI assistant accurately stated patient age in 94% of reports, and discrepancies were attributed to occurrence of birthday since initial workup. All 50 patients had a mammogram, with 86% (*n* = 43) receiving an ultrasound, and 36% (*n* = 18) having a breast MRI. The accuracy and completeness of the mammogram and ultrasound information was high (mammogram: 94% accurate and 92% complete; ultrasound: 86% accurate and 90.7% complete). Summarization by MRI was less often accurate (83.3%) or complete (44.4%). Among 85 biopsy reports, the biopsy modality was missing in 39.8% of the cases, with the AI-generated HPI stating “core biopsy” instead of specifying the type of imaging biopsy (i.e., stereotactic-guided biopsy). The accuracy of pathology reporting was high for histology (97.6%), receptor status (89.8%), and tumor grade (95%).

Among 12 patients requiring additional workup, 41.7% of the AI-generated HPIs appropriately identified pending studies. The overall clarity and relevance of the AI-generated HPIs were high, with average clarity rated 4.01 and relevance rated 3.87. Based on the initial model evaluation, the AI model was further modified to consistently include the biopsy modality and biopsy clip type in the HPI summaries before pilot testing.

### Pre-Survey on Documentation Practice

Of 18 team members, 15 respondents (83%) (9 PAs and 6 breast surgeons) completed the pre-survey regarding existing documentation practices for new patient consultations. Of these 15 respondents, 10 reported using a combination of templates plus free-text for assessment and plan documentation, and 5 used predominantly templated documentation. To document imaging and pathology reports (multiple answers were allowed in the survey), 10 (66.7%) free-text typed relevant portions, 6 (40%) copy-pasted the reports when possible, and 5 (33.3%) re-typed portions verbatim. With respect to timing of surgeon review, half of the surgeons reported reviewing the PA’s HPI summary before reviewing the chart, and the remaining half reported mixed review (before and after reviewing the chart).

### Pilot Testing

**Fig. 2:**
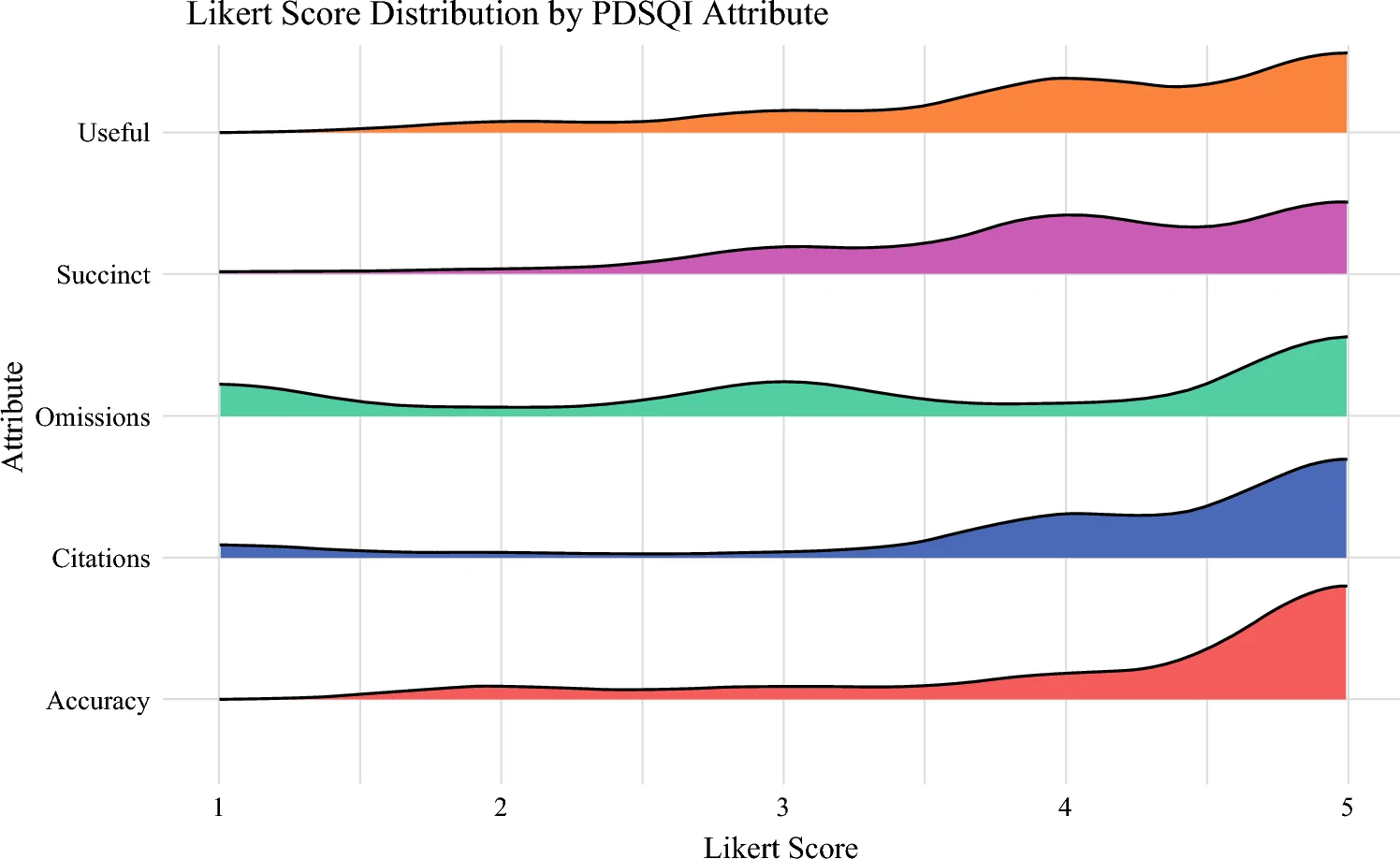
Density plot of Likert score distribution by PDSQI-9 attribute (*n* = 62). PDSQI-9, Provider Documentation Summarization Quality Instrument

The AI assistant was used in 62 sequential patient charts during the study period September 2025. Scoring for the AI summaries is detailed in Table 1. Overall, AI summaries scored high (Likert scores 4 or 5) in accuracy (84%), usefulness (81%), succinctness (79%), and in citing of the source documentation (85%). The AI summaries performed the worst in creating a thorough summary without omission of pertinent information (45% scored negatively). Among the summaries scoring below an average of 3 (*n* = 6), there were four instances of citation omission. Distribution of Likert scales by attribute tended to be skewed toward ratings of 4 or 5, but thoroughness without omission demonstrated a scattered distribution (Fig. 2).

**Table 1:** Provider documentation summarization quality instrument (PDSQI-9) average likert scores dichotomized by grouping with binomial testing (*p* < 0.05)

PDSQI-9 Question	Average Likert	High Score (Likert 4–5) *n* (%)	Low Score (Likert 1–3) *n* (%)	*p* Value
Citations present and appropriate	4.3	53 (85)	9 (15)	<0.001
Summary accurate	4.5	52 (84)	10 (16)	<0.001
Summary thorough and without omissions	3.6	34 (55)	28 (45)	0.526
Summary useful	4.2	50 (81)	12 (19)	<0.001
Summary succinct	4.2	49 (79)	13 (21)	<0.001

Comments regarding errors were reported in 25 (40%) surveys (Table 2). For example, 13 (52%) errors were classified as critical errors (e.g., no mention of a displaced biopsy clip, missing significant portions of imaging reports, incorrect laterality of breast cancer, and misrepresentation of axillary findings (e.g., borderline node listed as enlarged or axilla reported as negative when it had not been evaluated). The most common error was related to imaging, which was reported in 17 (68%) cases: 13 (52%) related to missing sections of reports, and 4 (16%) reporting inaccurate information such as mixing up the dates of studies, failure to mention clip or clip displacement, and axillary errors noted earlier. One respondent noted stigmatizing language, but details of this were not reported.

**Table 2:** Characterization of errors by type, frequency, and specific examples represented in the survey responses

Error Types	Error Examples
Critical errors (*n* = 13)	Failure to mention displaced biopsy clip, incorrectly labeled clip, absence of mammogram report and several MRI findings, omission of contralateral breast findings, incorrect laterality of breast cancer, misreporting of atypia as cancer, failure to note genetic variant
Organizational errors (*n* = 6)	Redundancy in summary, imaging/pathology reports noted to be pending but complete report present in PDF, omission of screening mammogram, pathology report listed under imaging section
Citation error (*n* = 4)	Citations omitted
Medical history error (*n* = 2)	Failure to mention current cardiac disease, history of cancer
Imaging error (*n* = 17)	13 with missing imaging information and 4 with inaccurate information, mostly missing screening mammograms, omission of laterality, incorrect dating, incomplete reports, one language error reported when a borderline lymph node was reported as enlarged, one reported axilla was negative but none of the studies had evaluated the axilla
Pathology error (*n* = 3)	Incorrect dates, completed reports listed as pending, patient with several biopsies unable to list which had FISH sent for HER2 testing, oncotype results missing, number of foci of microinvasion omitted
Breast cancer history error (*n* = 3)	Mode of detection incorrect (palpable vs. mammogram)
Genetics error (*n* = 2)	Failure to include a variant found on report, studies listed as pending when they had been completed

Error counts do not add up to total cases with errors as several cases included more than one type of error

### Post-Survey

The post-survey involved eight respondents, six PAs, and two surgeons. One PA reported always using the AI assistant, and the remaining seven respondents reported using it either “often” or “sometimes.” Half reported using the AI before they completed their own medical record review. One respondent used it after doing the review, and three reported mixed timing of use. The average NASA TLX for mental and temporal demand did not differ statistically compared with the pre-survey (mental: 9.3 versus 11; temporal: 8.5 versus 10.5; *p* > 0.05 for both).

Regarding the impact of the AI agent on time-saving, the Likert-scale scoring differed from the free-text responses. The median Likert response was 3 (mean, 2.9), with one respondent reporting a score of 4, and two reporting a score of 2. In the free-text response, the respondents described the AI agent as a “super helpful” and “good tool” and noted that it “saves time with actually typing out the findings but takes time to re-review and put in correct formatting and add/subtract things,” ultimately resulting in the chart preparation taking “about the same amount of time, maybe slightly less” than a manual review.

One respondent noted that “[a]dding this ‘proofing’ adds some time where I previously did not have this step when writing my own charts without the use of AI.” Three respondents noted that using it was an adjustment to their workflow (“great for straightforward cases . . . but needs improvement for more complex ones.” Another respondent “found that the content was great, but the formatting is so different from how (specific surgeon) writes her notes that it was quite time-consuming for me to convert.” Still another respondent provided specific feedback as to why it is not as effective for saving time, noting that “if it makes mistakes, this takes time to go in and fix—it can be a bit wordy and repetitive and I end up deleting/rearranging, which can end up taking more time than if I just typed it on my own . . . (e.g., changing punctuation, reordering things, fixing all the uppercase letters that the AI generates).”

## Discussion

This study demonstrated that AI-generated HPIs for breast cancer patient chart summarization have high clarity, relevance, and accuracy, and are clinically appropriate but frequently lack thoroughness and occasionally contain treatment-relevant errors. Although the tool was useful in minimizing typing of information from the PDF into the HPI summaries, specific formatting, including wordiness and repetitiveness, limited its usefulness.

Our pilot study did not definitively demonstrate a decrease in cognitive load by using the AI tool. This appeared to be driven by the need to correct errors in 40% of cases, lack of thoroughness of the summary in 45% of cases, workflow change, and clinicians’ preference for different HPI summary formats. Customizing the output to the clinician preference partly contributed to the workload.

Although there is growing enthusiasm and several commercially available products for decreasing documentation burden through AI-based chart summarization tools, understanding and measuring the actual value of AI tools in clinical practice and critically assessing how they fit into existing workflows before implementation are critical. To our knowledge, our study is the first of its kind to report the use of RAG-enabled GPT-4o to create pre-chart summarizations in breast surgical oncology when documentation is available only in PDF. This study was unique because it specifically aimed to understand the end-user’s perspective on the acceptability and feasibility of an AI summarization tool before widespread implementation.

Use of LLM-generated summaries have been tested in other settings. In primary care, AI-generated clinical summaries have been rated lower than clinician-authored ones but had less variability and were less likely to omit important information.^12^ In hospital course summarization, an EHR-embedded LLM produced summaries that required less editing than physician-generated ones.^13^ In emergency medicine, LLM-generated patient handoffs have been rated superior compared with physician-written summaries, but slightly inferior in usefulness.^14^

A study by Liang et al.,^15^ demonstrated the feasibility of GPT-4 in summarizing the EHR for new patient visits to a otolaryngology clinic for nasal congestion. Unlike our study, which focused on extraction of information from PDFs, that study used information within the EHR such as clinical notes and head and neck imaging studies. The study found that only 8% intended to use it without any edits, 67% intended to use it with edits, and 58% did not think the use of technology helped save time.^15^ These findings suggest that proofreading the AI’s summary can be a barrier to implementing these tools into practice because the factual inconsistencies are interwoven with accurate statements. As our results demonstrate, the “proofing” of the AI-generated HPI added a new step to which the clinical team was not accustomed, giving an overall neutral impression that the AI helps save time.

Given the 40% error rate in our study, with 52% of the errors classified as critical to treatment decisions, this “proofing” process also becomes critical to patient safety, further impacting the usefulness of the tool. Other studies examining time-saving with AI-generated summaries have demonstrated accuracy in helping clinicians answer patient-specific clinical questions, and with frequent AI-generated summary use, the clinicians were able to answer the questions faster.^16^ Also, in emergency medicine discharge summaries, use of an LLM assistant was associated with reduced writing time with similar quality.^17^

Other studies evaluating AI-generated summaries in breast cancer have demonstrated that oncologists favored AI-assisted (AI with human revision) over AI or human-only summaries.^18^ Thus, further iteration is required to improve the model’s output, which may ultimately translate to time-saving in pre-charting.

To facilitate the monitoring of the model’s performance, our AI model includes citations to the source documentation (patient PDF chart) in the generated HPIs. In four (6.5%) cases, the citations were not included by the AI, demonstrating potential drift of the model. The exact reason behind the loss of citations is not entirely clear, but growing evidence suggests that these AI tools are not static.^19^ The timing of our citation omission was clustered around an update to the system, and investigation is ongoing regarding the source of this error. This demonstrates an important need for additional studies to identify sustainable methods that evaluate for AI model drift. There have been developments in using LLMs as evaluators of LLMs, such as VeriFact, which has demonstrated 93.2% agreement with clinicians, higher than the interrater agreement among clinicians (88.5%), indicating that it can produce more consistent fact verification than humans.^20^

There are two potential benefits of AI-assisted technology, such as the one tested in our study. First, it has the potential to reduce documentation burden. Studies have observed an increase in message volume and outside of business hours EHR time for oncologists (e.g., ‘pajama time’),^21^ and that 44% of initial oncologic consultation duration is spent on EHR tasks, with fewer than one fourth of providers indicating that there is enough time for documentation.^22^ Technology such as ambient AI scribes has reduced documentation burden and physician burnout.^23^ However, current available AI scribe systems depend on ambient listening technology and do not address oncology-specific documentation needs based on review of radiology, pathology, and other oncology-specific reports. Thus, the AI scribe systems have the highest uptake in fields such as primary care and mental health that focus on direct patient-clinician communication for the encounter documentation.^24^

Second, AI-assisted extraction and summarization could facilitate formatting clinical data in an institutionally standardized format to ensure that all pertinent data are documented. Large language models can transform clinical data from unstructured to structured formats with high accuracy.^25^ Standardized documentation can save time for clinicians and facilitate patient care and extraction of information (e.g., tumor registry, research)^26,27^ and demonstrate greater clarity for recall.^28,29^

This study was not without limitations. In this single-institution evaluation, because of changes in the workforce during the pilot phase, not all the respondents who completed the pre-survey were available to participate in the post-survey. In addition, the model was developed specifically for breast surgical oncology using exclusively PDF records. However, following a similar development process and customization, this study would be generalizable to other cancers and specialties. Future studies will evaluate the scalability of this model and its impact on clinician satisfaction and downstream clinical outcomes such as visit efficiency and time to treatment.

## Conclusion

This study demonstrated the feasibility and acceptability of a RAG-enabled GPT-4o system in summarizing pertinent breast surgical oncology information. Our findings demonstrate that AI can be used for summarization of oncology documentation with high accuracy. Human oversight remains essential to ensure that critical information is not omitted, particularly for complex imaging details, biopsy modality, and pending workup. For success, AI-assisted oncology pre-charting will need further iterative refinement such as adding customization for clinicians, monitoring for model drift, fixing critical errors, and ensuring smooth workflow integration.

## Supplementary Information

Below is the link to the electronic supplementary material.

Supplementary file1 (PDF 203 KB)
