# Supplementary material for: Artificial Intelligence-Generated Electronic Medical Record Summarization in Breast Surgical Oncology
Source: Ann Surg Oncol. 2026 Jul 15;33(10):9102–9. doi: 10.1245/s10434-026-20170-w (PMC13585955; doi:10.1245/s10434-026-20170-w)
Supplement: Supplementary file 1 [file 10434_2026_20170_MOESM1_ESM.pdf]

Supplemental File

Modified Provider Documentation Summarization Quality Instrument (PDSQI-9) [5 point Likert scale; 1= Not at all, 5=Extremely]

1. Are citations present and appropriate? ("assertion" refers to statements made by the AI assistant in the HPI summary)

**Rating**	**Description**
1	Multiple incorrect citations OR No citations provided
2	One citation incorrect OR citations grouped together and not with individual assertions
3	Citations correct but some assertions missing a citation
4	Every assertion correctly cited with some relevance prioritization)
5	Every assertion is correctly cited and prioritized by relevance

2. Is the summary accurate in extraction?

**Rating**	**Description**
1: Not at All	Multiple major errors with overt falsifications or fabrications
2	A major error in assertion occurs with an overt falsification or fabrication
3	There is at least one assertion that is accurate but placed in the wrong context e.g. the correct US result placed incorrectly in the pathology section (At least one assertion contains a misalignment that is stated from a source note but the wrong context, including incorrect specificity in diagnosis or treatment)
4	There is at least one assertion that is accurate but included with unrelated information e.g. correct US result but with the incorrect date listed (At least one assertion is misaligned to the provider's source or timing but still factual in diagnosis, treatment, etc.)
5: Extremely	All assertions are accurate (All assertions can be traced back to the notes)

3. Is the summary thorough without any omissions?

**Rating**	**Description**
1	More than one pertinent omission occurs
2	One pertinent and possibly additional relevant details are left out (One pertinent and multiple potentially pertinent occur)

3	Only one pertinent omission occurs
4	Some potentially pertinent omissions occur
5	No pertinent or potentially pertinent omission occur

4. Is the summary useful?

Rating	Description
1: Not at All	The summary provides no useful information (No assertions are pertinent to the target user)
2	The summary includes some useful information (Some assertions are pertinent to the target user)
3	The summary includes useful information but is missing important details (Assertions are pertinent to target provider but level of detail inappropriate)
4	The summary only includes useful or potentially pertinent assertions (Not adding any non-pertinent assertions but some assertions are potentially pertinent to target user)
5: Extremely	The summary only provides useful information (Not adding any non-pertinent assertions and level of detail is appropriate to targeted user)

5. Is the summary succinct with economy of language?

Rating	Description
1: Not at All	Too many words and phrases are redundant/ repeated (Too wordy across all assertions with redundancy in syntax and semantics)
2	More than one assertion is redundant/repeated (More than one assertion has contextual semantic redundancy)
3	At least one assertion is repeated (At least one assertion has contextual semantic redundancy or multiple syntactic assertions)
4	No repeated assertions but at least one could be more succinct (No syntax redundancy in assertions and at least one could have been shorter in contextualized semantics)
5: Extremely	All assertions are captured with fewest words possible and without any redundancy in syntax or semantics

6. Is there stigmatizing language?
- yes/no

7. [free text] Describe any major errors from Breast Surgery Intake AI assistant output:
